# From cardio-oncology guidance to oncology nursing practice: A narrative review and preliminary practice map

**DOI:** 10.1016/j.apjon.2026.101050

**Published:** 2026-10-27

**Authors:** Chen Chen, Wenjiao Shan, Jingjing Hu, Yuankai Li, Yiqing Wang, Jiechun Huang, Xiaoli Yang

**Affiliations:** a Department of Cardiothoracic Surgery, Huashan Hospital, Fudan University, Shanghai, China; b Department of Oral and Maxillofacial–Head and Neck Surgery, Huashan Hospital, Fudan University, Shanghai, China; c Department of Neurosurgery, Huashan Hospital, Fudan University, Shanghai, China; d Department of Infection Control, Huashan Hospital, Fudan University, Shanghai, China

**Keywords:** Oncology nursing, Cardio-oncology, Narrative review, Implementation, Quality indicators, Survivorship

## Abstract

This narrative review examined how cardio-oncology guidance and related evidence can inform oncology nursing practice. Structured searches covered PubMed, Embase, and CINAHL Complete, and source selection was finalized through author review and consensus. The Scale for the Assessment of Narrative Review Articles informed reporting. Clinical guidance defined care tasks, while five publications directly addressed nursing roles, service models, learning needs, or education. Multidisciplinary care-delivery and service-implementation evidence described care-delivery gaps and service-level barriers. The synthesis distinguished these evidence contributions and organized them into an author-proposed preliminary practice map spanning five care stages from pretreatment assessment to survivorship follow-up. The map links proposed nursing contributions to the next responsible clinician or team, locally governed escalation, and six candidate nursing-relevant process measures. It is intended to support local workflow review, role negotiation, identification of training priorities, and feasibility auditing. The map remains unvalidated and requires stakeholder review, local adaptation, pilot testing, and prospective evaluation before broader use.

## Graphical abstract

**Graphical abstract.** Proposed nursing contributions across five cardio-oncology care stages include verification, symptom elicitation and education, protocol-based notification, coordination, and documentation and communication. Clinical decisions remain with authorized clinicians or teams. The preliminary map requires local adaptation, feasibility testing, and prospective evaluation.

**Figure:**
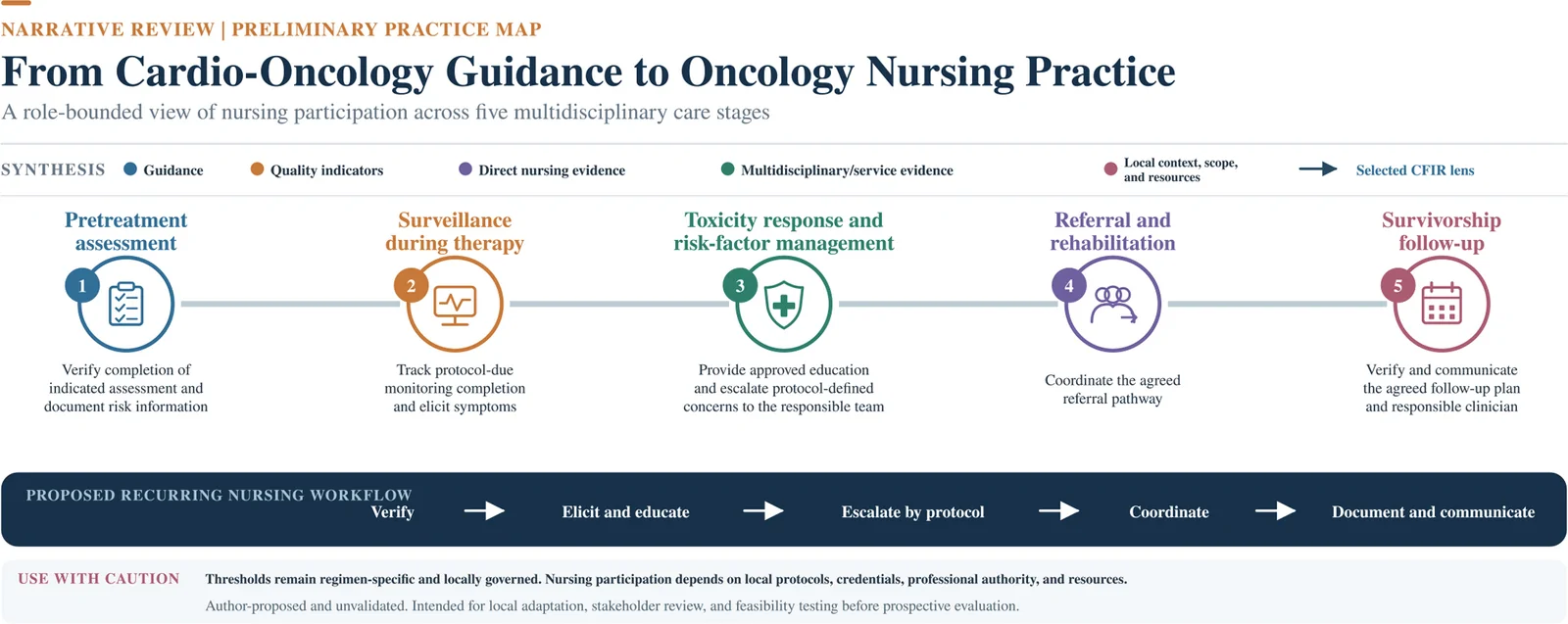

## Introduction

Worldwide, an estimated 20 million new cancer cases and 9.7 million cancer deaths occurred in 2022.^1^ Population-based research identifies cardiovascular mortality as an important burden among people diagnosed with cancer.^2^ In a community-based cohort of adult-onset cancer survivors, cardiovascular disease (CVD) risk varied by cancer type and risk-factor burden, and incident CVD was associated with poorer overall survival.^3^ These findings support attention to cardiovascular care, within the limits of the populations studied.

Detailed recommendations cover pretreatment cardiovascular risk assessment, surveillance during therapy, management of cancer therapy-related cardiovascular toxicity (CTR-CVT), referral, and post-treatment follow-up.^4^, ^5^, ^6^ Direct nursing publications describe potential contributions at repeated clinical touchpoints, including verification of planned care, symptom elicitation, education, coordination, and follow-up.^7^, ^8^ These activities occur within multidisciplinary pathways. Test ordering, clinical interpretation, treatment decisions, and independent referral remain governed by professional authority and local protocols.

This review addresses a practical problem: relevant recommendations and evidence are dispersed across clinical guidance, nursing publications, care-delivery studies, service reports, and formal quality-indicator work.^9^, ^10^ This distribution makes nursing touchpoints and handoffs difficult to translate into an auditable local workflow. The objective was to synthesize what clinical guidance, direct nursing evidence, multidisciplinary care-delivery evidence, and service-implementation evidence each contribute, then organize these distinct inputs into an author-proposed preliminary practice map. Asia–Pacific evidence was used to examine adaptation and transferability, not to imply a separate regional synthesis or region-wide applicability.

## Methods

### Review design and reporting

A narrative review was appropriate because the question required interpretive integration of heterogeneous clinical guidance, quality indicators, observational care-gap studies, nursing role and education publications, service-implementation reports, and conceptual frameworks. The purpose was neither to estimate a pooled effect nor to provide an exhaustive evidence map. The Scale for the Assessment of Narrative Review Articles (SANRA) informed reporting of the review rationale, objective, search, referencing, scientific reasoning, and relevant data.^11^ It was not used as an eligibility instrument, a risk-of-bias tool for included sources, or proof of systematic completeness.

### Information sources and search

PubMed, Embase (Ovid), and CINAHL Complete (EBSCOhost) were searched on August 25, 2026. PubMed and CINAHL were limited to English-language records published from January 1, 2015, through July 31, 2026. Embase permitted a 2015–2026 year limit, and the July 31 cutoff was applied during source selection. Search concepts covered cardio-oncology or cardiotoxicity, nursing or implementation and care processes, and geographic or resource context. Database-specific strategies and search-line results are reported in Supplementary Table S1A–S1C. The Embase table retains the original search-history numbering and identifies the lines contributing to the final export.

The exports contained 7972 records: 4659 from PubMed, 2672 from Embase, and 641 from CINAHL. Automated identifier and normalized-title de-duplication retained 6459 records. Additional duplicate and alternate-version review removed 40 records, leaving 6419 for title–abstract relevance classification. Reconciliation of the raw exports with the retained workbooks reproduced these counts. Chen, Wenjiao Shan, and Jingjing Hu manually reviewed the additional duplicate and alternate-version decisions. Item-level human sign-off for this step was not retained.

Thirty of the final 42 references were matched to records in the 6419-record database pool. Three pre-2015 foundational sources were documented as entering through citation checking to define implementation outcomes, hybrid study designs, and long-term survivorship risk.^12^, ^13^, ^14^ Nine final references, comprising guidance, epidemiological background, methodological or appraisal sources, and systematic reviews, did not match the database pool. The retained records do not allow their precise retrieval route to be distinguished reliably among prior-manuscript retention, citation checking, and targeted searching. They are therefore reported as route not documented rather than assigned retrospectively.

### Eligibility and source selection

Eligible sources addressed at least one of the following: cardio-oncology guidance or quality frameworks, delivery of recommended cardiovascular care, nursing roles or education, service models, referral, rehabilitation, survivorship, digital workflow, implementation determinants, or health-system and resource considerations relevant to adaptation. We excluded records without substantive cancer-related cardiovascular care content, preclinical or mechanistic studies without care-delivery relevance, diagnostic performance studies without an implementation question, case reports without transferable practice information, and conference abstracts with insufficient information. Sources were not required to use a particular study design. Operational criteria are detailed in Supplementary Table S2. Source-level accounting is provided in Supplementary Table S3A and S3B.

Chen, Wenjiao Shan, and Jingjing Hu independently assessed a 100-record sample to calibrate their application of the prespecified relevance categories. The authors then manually classified all 6419 records using these categories. Directly relevant records addressed a review objective or candidate map component. Contextual or indirect records informed background, determinants, or transferability. Full-text clarification records had potentially relevant abstracts but insufficient information for classification. Outside-scope records did not meet the operational criteria. These categories prioritized source review and were not binding inclusion decisions.

Chen selected candidate sources, including clarification records likely to fill a defined evidence role or resolve uncertainty. Wenjiao Shan and Jingjing Hu reviewed the candidates and confirmed the final cited set. Selection considered directness, incremental contribution, recency, treatment diversity, nursing relevance, and adaptation. Differences were resolved by discussion to consensus. The original reassessment reasons for five cited sources whose preliminary category differed from final retention were not retained.

Bibliographic details of the 42 cited sources were checked against PubMed records. Bibliographic verification was distinguished from assessment of whether the available source content supported the corresponding statements. An additional source-level verification exercise examined 18 candidates, comprising 17 full texts and one publisher abstract. The Medicare analysis by Huang et al. was examined through the publisher abstract, and the full-text Ivanescu survey was not retained. Supplementary Table S3C identifies the candidates, the material examined, and their final retention status. Supplementary Table S3B reports the material used for the documented claim-support checks and the scope of each citation. Supplementary Table S3D records the preliminary categories and current evidence roles of the five reassessed sources. Supplementary Table S4 presents the SANRA reporting-domain mapping.

### Synthesis and construction of the preliminary practice map

Chen drafted the interpretive mapping and the six candidate measures. Wenjiao Shan and Jingjing Hu reviewed every item, and differences were resolved by discussion to consensus. Construction proceeded in six steps. First, the five care stages were anchored to the 2026 International Cardio-Oncology Society (IC-OS)/Multinational Association of Supportive Care in Cancer (MASCC) statement and the 2022 European Society of Cardiology (ESC) guideline.^4^, ^6^ Second, evidence was classified as clinical guidance or formal quality guidance, direct nursing evidence, multidisciplinary clinical, care-delivery, service, or implementation evidence, or author-proposed operational translation. Third, selected Consolidated Framework for Implementation Research (CFIR) constructs were used as interpretive lenses.^15^ Fourth, each proposed nursing contribution was paired with the next responsible clinician or team and a locally governed escalation boundary. Fifth, every component was assigned exact reference numbers and a provenance label. Sixth, candidate measures were specified with an eligible population, numerator, denominator, period, data source, and interpretation. Established quality-indicator literature supplied measurement logic, while the six individual specifications were proposed by the authors.^9^, ^10^, ^12^ Supplementary Tables S5 and S6 provide stage-level and measure-level provenance.

CFIR constructs were selected qualitatively against three criteria: relevance, actionability, and applicability across care stages. Relevance referred to correspondence with a determinant or workflow question identified in the evidence synthesis. Actionability referred to whether a construct could inform a proposed local strategy or observable process. Applicability across care stages was considered at the level of the selected construct set, which was intended to support consistent mapping across the five stages. An individual construct was not required to appear at every stage. The mapping sequence was a reported gap or an explicitly author-proposed workflow concern, selected determinant, proposed strategy, role-bounded nursing contribution, next responsible clinician or team, local escalation boundary, and candidate measure. A worked surveillance example is presented in the section “CFIR-informed determinants and implementation strategies” after the corresponding evidence synthesis. No expert consensus exercise, stakeholder consultation, local workflow observation, pilot implementation, psychometric testing, or formal validation was undertaken.

## What existing publications provide and the narrower added value of this review

The five stages intentionally resemble the 2026 IC-OS/MASCC statement because they describe the same clinical continuum. The 2024 review by Lee and Reehal and the UK specialist-nurse report by Reehal et al. already describe baseline assessment, surveillance, education, coordination, referral, and long-term follow-up as potential nursing contributions.^6^, ^7^, ^8^ The Heart Failure Association–International Cardio-Oncology Society (HFA-ICOS) tool supplies structured baseline risk categories.^16^ Formal ESC quality indicators and a subsequent IC-OS/American College of Cardiology (ACC) roadmap provide population, numerator, denominator, measurement-period, and performance-measure principles.^9^, ^10^ Table 1 compares these source roles.

**Table 1:** Direct comparison with existing clinical, nursing, and quality publications.

Publication	Primary purpose	What it already provides	What the present review adds and does not add
Dent et al., 2026 (ref. 6)	Minimum clinical practice statement	Clinical tasks across five stages of the cancer trajectory	Uses the stages as clinical anchors. Does not add recommendations or thresholds.
Lee and Reehal, 2024, and Reehal et al., 2025 (refs. 7, 8)	Direct nursing role review and specialist service report	Potential nursing contributions and one specialist UK model	Makes role boundaries, handoffs, and evidence provenance explicit. Does not claim the activities are novel or universally transferable.
Lee et al., 2023, and Rao et al., 2025 (refs. 9, 10)	Formal quality indicators and measurement roadmap	Eligible populations, numerators, denominators, periods, and quality-measure principles	Uses the measurement logic for six unvalidated nursing-relevant process specifications. Does not replace formal indicators.
Present narrative review	Author-proposed preliminary practice map	Integrates distinct evidence categories	Links evidence source, care stage, determinant, proposed nursing contribution, next responsible actor, escalation boundary, and candidate process.

The strength-of-evidence analysis of the 2022 ESC guideline requires an explicit denominator. Five of 156 Class I recommendations carried Level of Evidence A, which is 3.2%. The same five represent 1.8% of all 272 recommendations.^17^ These values describe the evidence supporting recommendations, not clinical adherence.

## Evidence synthesis

### Patient-care and surveillance evidence

Historical studies identified incomplete baseline or serial cardiac assessment, mainly in breast cancer populations receiving trastuzumab or chemotherapy.^18^, ^19^, ^20^, ^21^, ^22^, ^23^ In the Australian cohort, baseline assessment occurred in 4984 of 5621 patients, or 88.7%. Guideline-recommended assessment, defined as baseline assessment plus on-treatment assessment at least every 120 days, occurred in 2702 of 4280 patients with at least 12 months of follow-up, or 63.1%.^21^ These studies identify pathway failure points, but they predate the 2022 ESC guideline or represent selected treatments and settings.

Evidence on response to detected toxicity and risk-factor support is also treatment-specific. Among 2625 anthracycline-treated patients, 226 developed cardiotoxicity. Following heart-failure therapy, 11% achieved full recovery and 71% partial recovery, with better recovery after earlier treatment.^24^ Medication adherence, exercise, and rehabilitation sources support attention to risk-factor management, but they do not validate a nursing-led pathway.^25^, ^26^, ^27^

A missed assessment or delayed response is a multidisciplinary care-delivery event, not evidence of nursing failure by itself. Ordering, access, clinical interpretation, treatment decisions, and referral acceptance depend on clinicians and organizations. Informed by direct nursing role literature,^7^, ^8^ this author-proposed map limits nursing contributions to checking completion, eliciting symptoms, providing approved education, coordinating agreed actions, documenting, and communicating concerns.

### Direct nursing evidence

Four publications directly addressed nursing roles, specialist nursing services, or learning needs.^7^, ^8^, ^28^, ^29^ A fifth direct nursing publication, the Cardio-Oncology Nursing Education (CONE) program description, is discussed with implementation resources below. Lee and Reehal described potential contributions of oncology nurses to assessment, surveillance, education, referral, and follow-up.^7^ Reehal et al. described a specialist clinical nurse role within a mature cardio-oncology service in the United Kingdom.^8^ Both are informative for role design, but neither tests the present map or establishes that general nurses can assume specialist or prescriber functions across jurisdictions.

Two surveys identified education needs. In an international English-language convenience survey, 268 of the 305 respondents who answered the relevant item, or 88%, reported no formal cardio-oncology education. The survey received 329 responses in total.^28^ A survey of 736 oncology nurses in tertiary hospitals in Shanxi Province found low knowledge scores using a stringent all-items-correct method.^29^ These findings support local education-needs assessment, but they do not demonstrate the effectiveness of a specific nursing intervention or permit region-wide prevalence estimates.

### Service, resource, treatment-diverse, and digital evidence

Contemporary service evidence remains heterogeneous. Repeated surveys of Japanese gynecologic oncologists found greater cardio-oncology awareness, while most respondents in both surveys reported no post-treatment cardiovascular testing. Response rates were low.^30^ A German hospital survey reported variation in risk assessment, monitoring, and multidisciplinary structures, but only 120 physicians from the 1435 invited departments responded.^31^ A recently published Medicare analysis used 2014–2018 data and therefore remains historical.^32^ Studies concerning immune checkpoint inhibitors, androgen-deprivation therapy, and anti-estrogen therapy reinforce the need for regimen-specific and indication–sensitive pathways rather than universal triggers.^33^, ^34^, ^35^

The single-center CFIR study was included to identify service-level determinants such as staffing, space, reporting lines, referral processes, imaging workflows, and electronic-record limitations.^36^ The electronic health record (EHR) trial was included because it tests a workflow support mechanism and distinguishes patient-reported discussion from recorded referral outcomes.^37^ Neither is direct nursing-effectiveness evidence, and neither establishes transferability to Asia–Pacific services. The CONE program describes 27 virtual modules and 18.25 contact hours, but patient and service outcomes have not been reported.^38^

Australian service mapping provides regional service context, while the China-focused commentary is used only as background on field development.^39^, ^40^ A Canadian telehealth review offers an external care-delivery model that may inform local questions about connectivity and access, but it does not test nursing implementation or establish Asia–Pacific transferability.^41^ Table 2 separates direct nursing evidence, patient-care and surveillance evidence, and service, resource, digital, or regional-context evidence. Direct nursing evidence supports possible touchpoints and education needs. Guidance defines clinical tasks. Care-delivery and service evidence identifies failure points and determinants. Specific workflow assignments and measure definitions remain author-proposed unless explicitly attributed.

**Table 2:** Evidence categories, potential nursing contributions, and multidisciplinary dependencies.

Evidence category and sources	Setting or design	Main contribution	Potential nursing contribution	Team or system dependency and limitation
***Direct nursing evidence: nursing roles and nursing education gaps***				
Direct nursing roles and service model (refs. 7, 8)	Review plus one specialist UK service report	Potential nursing touchpoints and specialist functions	May inform role discussion and locally authorized contributions	Does not validate the map or support universal transfer of specialist authority
Nursing learning needs (refs. 28, 29)	International convenience survey and Shanxi tertiary-hospital survey	Reported gaps in formal education or knowledge	Supports local education-needs assessment	Does not show intervention effectiveness or region-wide prevalence
Nursing education program (ref. 38)	Program description	Defined curriculum and contact hours	Offers a possible training resource	No patient, service, or implementation outcomes reported
***Patient-care and surveillance gaps***				
Historical surveillance studies (refs. 18, 19, 20, 21, 22, 23 and 32)	Mostly breast cancer, anti-HER2 therapy, chemotherapy, or older US data	Incomplete or variable baseline and serial assessment	Author-proposed verification of ordered care and notification of omissions, informed by nursing role literature (refs. 7, 8)	Ordering, access, interpretation, and treatment decisions are multidisciplinary or organizational
Toxicity and adherence evidence (refs. 24, 25) plus exercise and rehabilitation guidance (refs. 26, 27)	Prospective cohort, systematic review, consensus, and scientific statement	Earlier treatment may favor recovery, and risk support is relevant	Author-proposed symptom elicitation and approved education, informed by nursing role literature (refs. 7, 8)	Does not establish a nurse-led pathway or universal trigger
***Service, resource, digital, and regional barriers or context***				
Contemporary and treatment-diverse context (refs. 30, 31, 32, 33, 34, 35)	Surveys, claims, registry, expert recommendations, and cohort data	Persistent service variation and regimen-specific needs	Can inform local pathway tailoring	Professional, treatment, response-rate, and data-era limitations restrict inference
Service implementation (ref. 36)	Single-center CFIR mixed-methods study	Staffing, space, reporting, referral, imaging, and EHR barriers	Informs determinant and strategy discussion	Not nursing-specific and not directly transferable across systems
Digital workflow trial (ref. 37)	Practice-randomized EHR trial	Improved reported discussions but not recorded referral outcomes	Shows how prompts and outcome levels can be tested	Does not demonstrate nursing-specific or cardiovascular clinical benefit
Regional and external service-access context (refs. 39, 40, 41)	Australian service review, China commentary, and Canadian telehealth review	Metropolitan concentration, background on field development, and telehealth-related access considerations	Supports questions for local resource and access adaptation	Does not establish region-wide implementation, nursing performance, or direct transferability

Evidence categories are not interchangeable. Missed care does not establish a nursing-specific failure. CFIR, Consolidated Framework for Implementation Research; EHR, electronic health record; HER2, human epidermal growth factor receptor 2.

## CFIR-informed determinants and implementation strategies

The updated 2022 CFIR informed interpretation of the proposed workflow.^15^ For this interpretive use, the proposed locally adapted practice-map workflow was treated as the innovation rather than the underlying clinical regimens. Innovation Complexity concerned the scope, connections, and number of workflow steps. Compatibility concerned fit with existing workflows and treatment schedules. Available Resources concerned staff time and access to testing, while Access to Knowledge & Information concerned access to protocols and training. Local Conditions referred to relevant economic, environmental, political, or technological conditions. Financing, Partnerships & Connections, and Policies & Laws addressed reimbursement, referral networks, and scope-of-practice requirements, respectively. Planning concerned roles, responsibilities, steps, and measures. Engaging concerned participation in implementation and delivery. Reflecting & Evaluating concerned collecting and discussing information about implementation or workflow performance. These were author-selected operational interpretations, not a site-level CFIR assessment or a validated causal model.

The CFIR-guided mixed-methods study at one US safety-net center contributed a mechanism-level description of service barriers and informed the determinant-to-strategy mapping.^36^ Its 13 interviewees and 33 analyzable survey responses do not establish universal barriers or a nursing intervention effect.

The practice-randomized EHR trial contributed evidence about digital prompting and the difference between discussion, recommendation, and recorded referral.^37^ Among 627 evaluable survivors, the intervention increased patient-reported discussion of at least one cardiovascular factor and self-reported primary-care recommendation or referral, but it did not improve EHR-recorded referral outcomes or demonstrate cardiovascular clinical benefit. The study therefore informs workflow design and outcome selection, not nursing-specific effectiveness.

Surveillance during therapy provides a worked example of the authors' evidence-to-map translation. Guidance defines regimen- and risk-specific monitoring tasks.^4^, ^5^, ^6^ Selected studies in treatment-specific populations reported incomplete or delayed monitoring.^18^, ^19^, ^20^, ^21^, ^22^, ^23^, ^32^ These studies did not test CFIR determinants or establish the causes of the monitoring gaps. The authors applied Compatibility, Available Resources, and Planning as interpretive lenses concerning fit with treatment schedules, staff time and access to testing, and specification of due dates and responsibilities. The proposed strategy links monitoring due dates and, when applicable, cycle-linked reminders to treatment schedules. Direct nursing publications informed potential nursing touchpoints,^7^, ^8^ but reminders, the nursing verification assignment, notification timing, and measure specifications were author-proposed. Nurses, where authorized by local policy, would verify completion of ordered monitoring, document protocol-defined concerns, and notify the responsible clinician or team. P2 records whether protocol-due assessments are completed within the locally specified window and remains a team-delivered process that also depends on ordering, scheduling, access, and attendance. P2 and P3 are distinct endpoints. If a missed required assessment meets the locally defined criteria for an eligible escalation event, P3 records whether it is notified within the approved interval. P3 may also be triggered by protocol-defined symptoms or abnormal findings. Clinical review, interpretation, and treatment decisions remain with the responsible clinician or team.

## Preliminary oncology nursing practice map

The map is organized around five stages: pretreatment assessment, surveillance during therapy, toxicity response and risk-factor management, referral and rehabilitation, and survivorship follow-up. At each stage, Table 3 distinguishes a reported gap or author-proposed workflow concern, selected determinant and strategy, proposed nursing contribution, next responsible clinician or team, authority boundary, nursing-relevant process, and provenance. Supplementary Table S5 separates the sources supporting the clinical task, observed problem, potential nursing contribution, and author-proposed allocation. Fig. 1 summarizes construction and intended use. The **graphical abstract** outlines the recurring workflow across the five stages. Thresholds remain regimen-specific and locally governed.

**Table 3:** Evidence-to-action traceability for the preliminary oncology nursing practice map.

Care stage	Reported gap or proposed concern and type	Selected updated CFIR constructs and proposed strategy	Proposed nursing contribution	Next responsible clinician or team and authority boundary	Nursing-relevant process	Exact provenance
Pretreatment assessment	Indicated baseline assessment or risk information may be absent. Patient-care gap.	Innovation Complexity, Access to Knowledge & Information, Compatibility. Use a regimen-linked checklist.	Verify completion of ordered baseline cardiovascular assessments and document the cardiovascular risk information specified in the local protocol.	Responsible oncology prescriber or team reviews risk and decides testing, treatment, or referral. Independent action requires local authority.	P1 Baseline assessment status verification	G: refs. 4, 5, 6, 9 and 16. N: refs. 7, 8. S: refs. 18, 19, 20, 21, 22, 23 and 32. A: checklist, assignment, and handoff.
Surveillance during therapy	Protocol-due monitoring may be late, missed, or disconnected from treatment schedules. Patient-care and system gap.	Compatibility, Available Resources, Planning. Link regimen- and risk-specific due dates to treatment schedules.	Where assigned by local policy, track completion of protocol-due monitoring, elicit protocol-specified symptoms, document eligible concerns, and notify the responsible clinician or team.	Responsible oncology clinician or team interprets results and decides treatment. Urgent concerns follow the approved pathway.	P2 On-time protocol-due surveillance completion. P3 Timely protocol-defined notification.	G: refs. 4, 5, 6, 9. N: refs. 7, 8. S: refs.^18^, ^19^, ^20^, ^21^, ^22^, ^23^, ^32^ and 37. A: reminders, nursing endpoint, and timing.
Toxicity response and risk-factor management	Symptoms, abnormalities, or risk factors may not receive timely attention. Patient-care and education gap.	Access to Knowledge & Information, Engaging, Compatibility. Use structured review and approved education.	Elicit protocol-specified symptoms, provide approved education, document concerns, and notify the designated team.	Authorized oncology or cardio-oncology clinician assesses, diagnoses, and manages. Emergency symptoms follow emergency policy.	P3 Timely protocol-defined notification. P4 Cardiovascular education completion.	G: refs. 4, 5, 6, 26, and 27. N: refs. 7, 8. S: refs. 24, 25, and 33. A: review bundle and handoff.
Referral and rehabilitation	Indicated patients may encounter unclear routes or limited access. Service and resource gap.	Available Resources, Partnerships & Connections, Policies & Laws, Engaging. Define an authorized referral pathway and locally approved alternatives.	Communicate and coordinate an authorized referral, transmit required information, track status, and report barriers. Independent referral requires local authority.	Authorized clinician or team documents the referral decision. The receiving service determines acceptance and completes assessment. Alternatives require clinician approval.	P5 Timely referral transmission after an authorized decision	G: refs. 4, 5, 6, 27, and 34. N: refs 7, 8. S: refs. 31, 36, 37, 39 and 41. A: transmission workflow and endpoint.
Survivorship follow-up	Long-term CV risk persists, while care-plan benefits are uncertain. Risk and outcome context. Transition ownership is an author-proposed workflow concern.	Compatibility, Planning, Reflecting & Evaluating. Define minimum plan fields and ownership.	Verify that a team-agreed plan contains required fields, communicate it to the patient and next clinician, and document transmission.	The authorized oncology or cardiovascular team determines surveillance and management. The named clinician assumes follow-up responsibility.	P6 Survivorship-plan verification and communication	G: refs. 4, 5, 6. N: refs. 7, 8. S: refs. 14, 42. A: minimum fields, nursing verification, communication, and handoff.

Thresholds and time windows must follow applicable regimen-specific guidance or a locally approved protocol. Proposed nursing contributions do not transfer clinical accountability. G denotes clinical guidance or formal quality guidance, N direct nursing evidence, S multidisciplinary clinical, care-delivery, service, or implementation evidence, and A author-proposed operational translation. A provenance category does not imply that every source in it supports every component. Supplementary Table S5 gives component-specific attribution. CFIR means Consolidated Framework for Implementation Research. CV means cardiovascular.

**Fig. 1:**
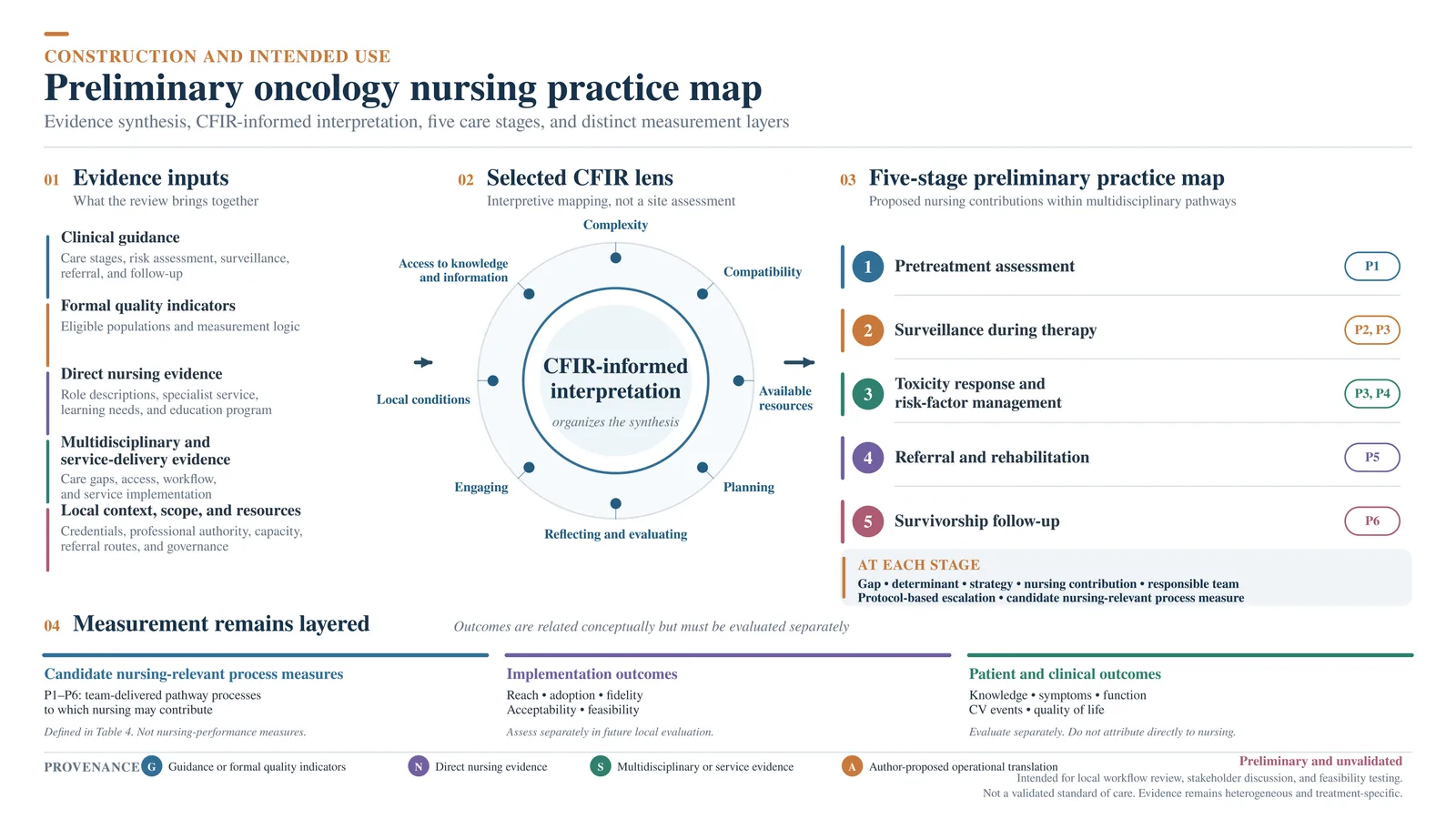
**Construction and intended use of the preliminary oncology nursing practice map.** Evidence and local context inform the five-stage map. The ring shows a subset of the selected updated CFIR constructs. Candidate process measures, implementation outcomes, and patient outcomes are distinguished. Table 3, Table 4 detail proposed nursing contributions, responsibility boundaries, and measure specifications. P3 records timely notification of protocol-defined concerns, and P5 records referral transmission. The map is unvalidated and intended for local workflow review and feasibility testing. CFIR, Consolidated Framework for Implementation Research; CV, cardiovascular.

### Role boundaries

Within this author-proposed map, and where assigned by local policy, a general oncology nurse may verify completion of ordered assessments, collect protocol-specified symptoms and observations, deliver approved education, document findings, and notify the designated team. Direct nursing role publications inform these potential touchpoints.^7^, ^8^ Clinical interpretation and decisions to start, delay, or modify treatment, order additional testing, or refer remain with the authorized clinician or multidisciplinary team. A specialist cardio-oncology nurse may undertake advanced assessment, coordination, education, audit, or follow-up where training and policy permit. An advanced practice nurse may order tests, prescribe, interpret results, or refer independently only where credentials, legislation, and institutional policy authorize those functions.^8^

The proposed nursing contributions draw on direct nursing role literature^7^, ^8^ but extend beyond what those publications directly tested. Table 3 therefore labels author-proposed allocations explicitly. Education remains an enabling strategy rather than evidence of clinical effectiveness. Local services should assess competency and authority rather than infer either from course completion.^28^, ^29^, ^38^

## Candidate measures and outcomes

Table 3, Table 4 serve different purposes. Table 3 describes proposed activities, handoffs, and responsibility boundaries. Table 4 specifies how selected team-delivered processes might be measured. P1–P6 are therefore termed candidate nursing-relevant process measures, not nursing-performance measures. They were selected because they represent recurrent processes at nursing touchpoints, are potentially actionable and recordable, apply across the five stages, and can be separated from downstream clinical decisions. Existing ESC indicators and quality-measure literature supplied population, numerator, denominator, timing, and data-source logic.^9^, ^10^ The individual endpoints and operational wording are author-proposed.

**Table 4:** Operational definitions for author-proposed candidate nursing-relevant process measures.

ID and endpoint	Eligible population	Numerator	Denominator	Assessment period	Primary data source	Interpretation and responsibility boundary
P1 Baseline assessment status verification	Treatment starts for which the current guideline or local protocol requires baseline CV assessment	Eligible starts with nursing documentation of a status for every ordered or locally required assessment element: completed, documented by the responsible clinician as not appropriate, or missing with notification to the responsible clinician or team	All eligible treatment starts	Before first dose, summarized monthly or quarterly	Treatment plan, checklist, orders, results, and EHR	Completeness of nursing verification, not completion of all assessments. Report the status of each assessment element separately, including mixed statuses within a treatment start. Clinical decisions remain with the responsible clinician.
P2 On-time protocol-due surveillance completion	Protocol-due assessments under regimen- and risk-specific rules	Due assessments completed within the locally specified window	All protocol-due assessments	Per due window, summarized monthly or quarterly	Treatment schedule, orders, results, and EHR	Timeliness of indicated monitoring. Completion depends on ordering, scheduling, access, and attendance.
P3 Timely protocol-defined notification of an eligible escalation event	Protocol-defined symptoms, missed required assessments, or abnormal findings first identified at a nursing touchpoint	Eligible events with documented notification to the named responsible clinician or team within the locally approved interval	All eligible notification events	Event level, summarized monthly or quarterly	Nursing note, task or inbox log, and communication record	Measures the nursing communication endpoint only. Completed clinical review and treatment decisions are separate team outcomes.
P4 Cardiovascular education completion	Education encounters required by the approved pathway at baseline, during treatment, or at transition	Eligible encounters with documented delivery of required topics and a comprehension or teach-back check	All eligible education encounters	At each designated stage, summarized quarterly	Education flowsheet, nursing note, or patient portal	Measures delivery and documentation. It does not prove sustained knowledge, behavior change, or clinical benefit.
P5 Timely referral transmission after an authorized decision	Referral decisions documented by an authorized clinician or team	Eligible decisions for which the referral order or request and required information were transmitted to the named receiving service within the locally specified interval	All documented authorized referral decisions	Event level, summarized monthly or quarterly	Decision record, referral order or request, transmission log, and scheduling system	Measures transmission, not acceptance or completion. Report acceptance, attendance, completion, access failure, and clinician-approved alternatives separately. An alternative is not a completed referral.
P6 Survivorship-plan verification and communication	Eligible transitions for which the responsible team has agreed an ongoing CV surveillance or risk-management plan	Eligible care transitions with documentation that nursing staff verified the required elements of the team-agreed survivorship plan and communicated the plan to the patient and receiving clinician	All eligible transitions with an agreed plan due	At transition, summarized quarterly	Survivorship plan, discharge summary, nursing note, and transmission log	Measures nursing verification and communication. It does not imply that the nurse independently determines surveillance requirements.

These team-delivered pathway measures are proposed for local testing. They are not endorsed quality indicators or measures of nursing performance. CV, cardiovascular; EHR, electronic health record.

Implementation outcomes should be distinguished from service-process and patient outcomes. Candidate implementation outcomes include penetration, adoption, fidelity, acceptability, feasibility, and sustainability.^12^ If reach is assessed, its target population and denominator should be specified separately. Time to completed clinical review is a service-process outcome. Patient and clinical outcomes include knowledge, self-efficacy, treatment interruption, cardiovascular events, symptoms, function, and quality of life. P3 measures timely notification, not completed clinical review. P5 measures referral transmission after an authorized decision, while acceptance, attendance, completion, and a clinician-approved alternative are reported separately. A documented alternative is not counted as a completed referral.

## Adaptation across Asia–Pacific health systems

This review included regional sources that differed in profession, disease, treatment, and service setting. They comprised a provincial survey of oncology nurses in Shanxi Province, China, repeated surveys of Japanese gynecologic oncologists, a Korean tertiary-hospital cohort, Australian population and service reports, Asia–Pacific prostate cancer recommendations, and a China-focused commentary used only as background context.^21^, ^23^, ^29^, ^30^, ^34^, ^39^, ^40^ They inform questions for local adaptation but do not establish applicability across Asia–Pacific health systems.

Local adaptation should consider nursing authority, staff competencies, specialist access, imaging and biomarker capacity, referral pathways, rural distance, language, connectivity, and continuity of follow-up. Australian services illustrate metropolitan concentration. Telehealth may support coordination where connectivity permits, but it does not remove the need for clinically indicated in-person assessment or imaging.^39^, ^41^ Findings from US safety-net and community oncology settings may suggest workflow mechanisms, but they require local appraisal before transfer.^36^, ^37^ In resource-constrained services, an initial feasibility pilot could focus on low-burden workflow supports, including an approved checklist, protocol-specified symptom and blood-pressure documentation, approved education, and a named escalation contact. These supports would supplement, not replace, referral or shared access to regimen- and risk-specific assessment, imaging, or biomarkers when clinically indicated.

Local evaluation could begin with one clearly defined treatment pathway at a single site. Local stakeholders can review proposed roles and authority boundaries, assess whether data required for one or two measures such as P1 and P2 are available, and use a short baseline audit to identify documentation, scheduling, access, or handoff gaps. The service can then adapt the workflow and conduct a small pilot focused on feasibility and unintended effects. Further details of staged validation and effectiveness evaluation are presented in the following section.

## Research and validation priorities

Subsequent evaluation should proceed in stages. Findings from stakeholder review and the initial feasibility pilot should be used to refine roles, workflow steps, data fields, and measures. Only after feasibility, acceptability, workload, data completeness, equity, and unintended effects have been examined and the workflow and measures are stable should a larger study examine effectiveness. Hybrid designs may assess implementation and clinical outcomes together while retaining the distinction between them.^13^

Future evidence should expand beyond breast cancer and cardiomyopathy to immune checkpoint inhibitors, androgen-deprivation therapy, cellular therapy, radiation, and lifelong treatment. Survivorship evaluation should not assume that delivery of a survivorship care plan improves outcomes because prior trials have shown uncertain clinical benefit.^42^ Long-term cardiovascular risk among childhood cancer survivors supports durable follow-up, but does not by itself establish a transition-of-care failure.^14^ The proposed plan-ownership and communication steps require local testing.

## Limitations

This narrative review has important limitations. Searches were limited to English-language records from 2015 onward, with three earlier foundational sources retained through documented citation checking. The original item-level retrieval route for nine final references and the original reassessment reasons for five category-to-retention transitions were not recoverable from retained records. Source checks and retention rationales documented during revision do not reconstruct those historical steps. Selection was purposive, and the preliminary manual relevance categories prioritized source review rather than constituting an exhaustive inclusion procedure. Care-gap evidence is heterogeneous and concentrated in selected cancers, treatments, professions, and health systems. Five publications directly addressed nursing roles, services, learning needs, or education, but none validated the map or demonstrated patient benefit from a nursing intervention. The source materials available for claim checking varied, and source-specific limits are reported in the supplement. CFIR interpretation, role allocations, and candidate measures were author-proposed without stakeholder consultation or validation.

## Conclusions

Clinical guidance defines the principal cardio-oncology tasks, direct nursing evidence identifies potential touchpoints and education needs, and multidisciplinary or service evidence identifies delivery barriers. This review organizes those distinct inputs into a five-stage preliminary map with proposed nursing contributions, named handoffs, locally governed escalation, exact provenance, and six candidate nursing-relevant process measures. Its intended use is local workflow review, role negotiation, training-priority identification, and feasibility auditing. It does not transfer multidisciplinary accountability to nurses or establish a validated standard. Stakeholder review, local adaptation, pilot testing, and prospective evaluation are required.

## CRediT authorship contribution statement

**Chen Chen:** Conceptualization, Methodology, Investigation, Data curation, Validation, Writing – original draft. **Wenjiao Shan:** Investigation, Data curation, Validation, Writing – review and editing. **Jingjing Hu:** Investigation, Data curation, Validation, Writing – review and editing. **Yuankai Li:** Writing – review and editing. **Yiqing Wang:** Methodology, Supervision, Writing – review and editing. **Jiechun Huang:** Conceptualization, Supervision, Writing – review and editing. **Xiaoli Yang:** Supervision, Project administration, Writing – review and editing. All authors have read and approved the final manuscript.

## Ethics statement

Not required.

## Data availability

No new patient-level data were generated or analyzed. Search exports, classification records, and source-verification records supporting the transparent narrative-review process are available from the corresponding author, subject to database licensing restrictions.

## Declaration of generative AI and AI-assisted technologies in the writing process

During the preparation of this work, the authors used Claude (Anthropic) to check grammar and spelling. After using this tool, the authors reviewed and edited the content as needed and take full responsibility for the content of the published article.

## Funding

This study received no external funding.

## Declaration of competing interest

The authors declare no conflict of interest.
