# Supplementary material for: From cardio-oncology guidance to oncology nursing practice: A narrative review and preliminary practice map
Source: Asia Pac J Oncol Nurs. 2026 Oct 27;13:101050. doi: 10.1016/j.apjon.2026.101050 (PMC13762020; doi:10.1016/j.apjon.2026.101050)
Supplement: Supplementary file 1 [file mmc1.docx]

Supplementary Methods and Evidence Traceability

From Cardio-Oncology Guidance to Oncology Nursing Practice: A Narrative Review and Preliminary Practice Map

This supplement describes the structured searches and purposive selection used in the narrative review, records the source checks documented during revision, and links the practice map and candidate measures to their sources. Supplementary Tables S1–S4 support the Methods. Supplementary Table S5 expands the component-level provenance in main-text Table 3, and Supplementary Table S6 explains the derivation of P1–P6 in main-text Table 4.

# Supplementary Methods

## Design and rationale

A narrative review was used to integrate clinical guidance, formal quality indicators, nursing literature, observational care-delivery studies, service reports, and implementation concepts. Structured searches supported purposive source selection. SANRA informed reporting. The review did not use a registered protocol, formal risk-of-bias assessment, or quantitative synthesis.

## Search date and window

All databases were searched on August 25, 2026. PubMed and CINAHL used a January 1, 2015, to July 31, 2026, publication-date limit. Embase was searched in a database updated through August 20, 2026, with a 2015–2026 year limit. The July 31 cutoff was applied during source selection.

## Source accounting

The exports contained 7,972 records: 4,659 from PubMed, 2,672 from Embase, and 641 from CINAHL. Automated de-duplication retained 6,459 records. Further duplicate and version checking removed 40 records, leaving 6,419 unique records. Chen Chen, Wenjiao Shan, and Jingjing Hu manually reviewed these decisions. Item-level human sign-off for this step was not retained. Thirty of the final 42 references matched this pool. Three pre-2015 sources entered through documented citation checking. The original retrieval route for nine final references was not documented in the retained records. This describes a gap in retrieval documentation, not evidence that their full texts were unavailable or unread. Table S3A records the counts, and Table S3B identifies each final source.

## Selection and classification

Chen Chen, Wenjiao Shan, and Jingjing Hu independently assessed a 100-record sample to calibrate their application of the prespecified relevance categories. The authors then manually classified all 6,419 records using these categories. Directly relevant records addressed a review objective or practice-map component. Contextual or indirect records informed background, determinants, or transferability. Full-text clarification records appeared relevant but could not be classified from the title and abstract. Outside-scope records did not meet the operational criteria. These categories prioritized source review and were not binding inclusion decisions. Chen Chen selected candidate sources, and Wenjiao Shan and Jingjing Hu reviewed them and confirmed the final cited set. Selection considered directness, incremental contribution, recency, treatment diversity, nursing relevance, and adaptation. Differences were resolved by discussion to consensus.

## Bibliographic and claim-support verification

Bibliographic identity and claim support were checked separately. PubMed matching confirmed the metadata of all 42 final references. The earlier additional source-level exercise examined 18 candidates, comprising 17 full texts and the publisher abstract for Huang et al. (ref. 32). Seventeen candidates were retained in the final reference set. The Ivanescu et al. survey was checked in full text but was not retained. Table S3C identifies all 18. Table S3B documents the source material and claim-specific checks recorded during revision. Source access and the original retrieval route are reported separately. Table S3D explains the current evidence roles of five retained sources whose preliminary categories did not establish final inclusion.

## Interpretive mapping and measure development

Chen Chen drafted the interpretive CFIR mapping, main-text Table 3, and the six candidate measures. Wenjiao Shan and Jingjing Hu reviewed every item, and differences were resolved by discussion to consensus. CFIR constructs were selected for relevance to the reported problem, potential to inform a practical response, and applicability across care stages. Clinical guidance defined the care requirements. Nursing literature informed nursing activities, while care-delivery and service studies identified problems and contextual constraints. The authors then linked these contributions to proposed workflows, responsible clinicians or teams, and candidate measures. Tables S5 and S6 distinguish established source contributions from the proposed combinations, handoffs, and measurement specifications. This was interpretive mapping rather than a site-level CFIR assessment.

**Supplementary Table S1A. PubMed search strategy, searched August 25, 2026.**

*Database: PubMed/MEDLINE. Language: English. Publication date: 2015-01-01 to 2026-07-31.*

| **Line** | **Search** | **Results** |
| --- | --- | --- |
| #1 | ("Cardio-Oncology"[MeSH Terms] OR ("Cardiotoxicity"[MeSH Terms] AND ("Neoplasms"[MeSH Terms] OR "Antineoplastic Agents"[MeSH Terms])) OR cardio-oncolog*[Title/Abstract] OR cardiooncolog*[Title/Abstract] OR CTRCD[Title/Abstract] OR ((cancer*[Title/Abstract] OR oncolog*[Title/Abstract] OR neoplas*[Title/Abstract] OR antineoplastic*[Title/Abstract] OR anticancer[Title/Abstract] OR anti-cancer[Title/Abstract] OR chemotherap*[Title/Abstract] OR immunotherap*[Title/Abstract] OR radiotherap*[Title/Abstract]) AND (cardiotoxic*[Title/Abstract] OR "cardiac toxicity"[Title/Abstract] OR "cardiovascular toxicity"[Title/Abstract] OR "cardiac dysfunction"[Title/Abstract]))) | 17,954 |
| #2 | ("Oncology Nursing"[MeSH Terms] OR "Nursing Care"[MeSH Terms] OR "Advanced Practice Nursing"[MeSH Terms] OR "Nurse's Role"[MeSH Terms] OR "Implementation Science"[MeSH Terms] OR "Guideline Adherence"[MeSH Terms] OR "Quality Indicators, Health Care"[MeSH Terms] OR "Quality Improvement"[MeSH Terms] OR "Outcome and Process Assessment, Health Care"[MeSH Terms] OR "Referral and Consultation"[MeSH Terms] OR "Rehabilitation"[MeSH Terms] OR "Patient Education as Topic"[MeSH Terms] OR "Clinical Competence"[MeSH Terms] OR "Continuity of Patient Care"[MeSH Terms] OR "Risk Assessment"[MeSH Terms] OR nurs*[Title/Abstract] OR "advanced practice"[Title/Abstract] OR implement*[Title/Abstract] OR adherence[Title/Abstract] OR (guideline*[Title/Abstract] AND concordan*[Title/Abstract]) OR (quality[Title/Abstract] AND (indicator*[Title/Abstract] OR measure*[Title/Abstract] OR improvement[Title/Abstract])) OR "care pathway"[Title/Abstract] OR "care pathways"[Title/Abstract] OR "care gap"[Title/Abstract] OR "care gaps"[Title/Abstract] OR "service model"[Title/Abstract] OR "service models"[Title/Abstract] OR (survivorship[Title/Abstract] AND plan*[Title/Abstract]) OR referral*[Title/Abstract] OR rehabilitat*[Title/Abstract] OR educat*[Title/Abstract] OR competenc*[Title/Abstract] OR monitor*[Title/Abstract] OR surveillance[Title/Abstract] OR "risk assessment"[Title/Abstract] OR "risk assessments"[Title/Abstract] OR "care coordination"[Title/Abstract] OR follow-up[Title/Abstract] OR "follow up"[Title/Abstract]) | 7,522,422 |
| #3 | ("Asia"[MeSH Terms] OR "Oceania"[MeSH Terms] OR "Rural Health Services"[MeSH Terms] OR "Rural Population"[MeSH Terms] OR "Medically Underserved Area"[MeSH Terms] OR "Developing Countries"[MeSH Terms] OR Asia[Title/Abstract] OR Asian[Title/Abstract] OR "Asia Pacific"[Title/Abstract] OR "Asia-Pacific"[Title/Abstract] OR Oceania*[Title/Abstract] OR Australasia*[Title/Abstract] OR China[Title/Abstract] OR Chinese[Title/Abstract] OR "Hong Kong"[Title/Abstract] OR Taiwan[Title/Abstract] OR Japan*[Title/Abstract] OR Korea*[Title/Abstract] OR India*[Title/Abstract] OR Singapore*[Title/Abstract] OR Malaysia*[Title/Abstract] OR Indonesia*[Title/Abstract] OR Philippine*[Title/Abstract] OR Filipino[Title/Abstract] OR Thailand[Title/Abstract] OR Thai[Title/Abstract] OR Vietnam*[Title/Abstract] OR Pakistan*[Title/Abstract] OR Bangladesh*[Title/Abstract] OR "Sri Lanka"[Title/Abstract] OR Nepal*[Title/Abstract] OR Australia*[Title/Abstract] OR "New Zealand"[Title/Abstract] OR "Pacific Island"[Title/Abstract] OR "Pacific Islands"[Title/Abstract] OR "Pacific Islander"[Title/Abstract] OR "Pacific Islanders"[Title/Abstract] OR rural[Title/Abstract] OR "low resource"[Title/Abstract] OR "low-resource"[Title/Abstract] OR "resource limited"[Title/Abstract] OR "resource-limited"[Title/Abstract]) | 2,827,881 |
| #4 | #2 OR #3 | 9,442,329 |
| #5 | #1 AND #4 | 6,506 |
| #6 | #5 AND english[Language] AND ("2015/01/01"[Date - Publication] : "2026/07/31"[Date - Publication]) | 4,659 |

**Supplementary Table S1B. Embase (Ovid) search strategy, searched August 25, 2026.**

*Database coverage displayed by Ovid: 1974 to 2026 August 20. Final export: line 18, n=2,672. The July 31 cutoff was applied during source selection because the interface used a calendar-year limit. Original Ovid search-history line numbers are retained. The final export depends on lines 4, 10, 11, and 15–18. All expressions needed to reconstruct that combination are reproduced below.*

| **Line** | **Search** | **Results** |
| --- | --- | --- |
| 1 | exp cardiotoxicity/ or exp cardio-oncology/ | 64,200 |
| 2 | (cardio-oncolog* or cardiooncolog* or cardiotoxic* or (cancer therap* adj3 cardiovascular toxic*)).ti,ab,kw. | 38,523 |
| 3 | 1 or 2 | 74,374 |
| 4 | (nurs* or advanced practice or implement* or adherence or guideline concordan* or quality indicator* or quality measure* or quality improvement or care pathway* or care gap* or service model* or survivorship care plan* or referral* or rehabilitation or education or competenc*).ti,ab,kw. | 3,676,908 |
| 10 | (Asia or Asian or (Asia adj1 Pacific) or Oceania* or Australasia* or China or Chinese or Hong Kong or Taiwan or Japan* or Korea* or India* or Singapore* or Malaysia* or Indonesia* or Philippine* or Thailand or Vietnam* or Pakistan* or Bangladesh* or Sri Lanka* or Nepal* or Australia* or (New adj1 Zealand*) or (Pacific adj1 Island*) or rural or low-resource or resource-limited).ti,ab,kw. | 2,953,513 |
| 11 | 4 or 10 | 6,182,608 |
| 15 | exp cardio-oncology/ or (exp cardiotoxicity/ and (exp neoplasm/ or exp antineoplastic agent/)) or (cardio-oncolog* or cardiooncolog* or CTRCD or ((cancer* or oncolog* or neoplas* or antineoplastic* or anticancer or chemotherap* or immunotherap* or radiotherap*) adj5 (cardiotoxic* or cardiac toxic* or cardiovascular toxic* or cardiac dysfunction))).ti,ab,kw. | 46,746 |
| 16 | 15 and 11 | 3,678 |
| 17 | limit 16 to English language | 3,549 |
| 18 | limit 17 to yr="2015 - 2026" | 2,672 |

**Supplementary Table S1C. CINAHL Complete (EBSCOhost) search strategy, searched August 25, 2026.**

*Fields: title, abstract, and CINAHL Headings. Search mode: proximity search with equivalent subjects enabled. Final filtered export: n=641.*

| **Line** | **Search** | **Results** |
| --- | --- | --- |
| S1 | cardio-oncology (mapped term/keyword, “Apply equivalent subjects” enabled) | 547 |
| S2 | MH "Cardiotoxicity" | 1,991 |
| S3 | MH "Neoplasms+" OR MH "Antineoplastic Agents+" | 745,660 |
| S4 | S2 AND S3 | 1,510 |
| S5 | TI ((cardio N1 oncolog*) OR cardiooncolog* OR CTRCD OR ((cancer* OR oncolog* OR neoplas* OR antineoplastic* OR anticancer OR chemotherap* OR immunotherap* OR radiotherap*) N5 (cardiotoxic* OR "cardiac toxicity" OR "cardiovascular toxicity" OR "cardiac dysfunction"))) OR AB ((cardio N1 oncolog*) OR cardiooncolog* OR CTRCD OR ((cancer* OR oncolog* OR neoplas* OR antineoplastic* OR anticancer OR chemotherap* OR immunotherap* OR radiotherap*) N5 (cardiotoxic* OR "cardiac toxicity" OR "cardiovascular toxicity" OR "cardiac dysfunction"))) | 1,802 |
| S6 | S4 OR S5 | 2,692 |
| S7 | TI (nurs* OR "advanced practice" OR implement* OR adherence OR (guideline N1 concordan*) OR (quality N1 indicator*) OR (quality N1 measure*) OR (quality N1 improvement) OR (care N1 pathway*) OR (care N1 gap*) OR (service N1 model*) OR (survivorship N3 plan*) OR referral* OR rehabilitat* OR educat* OR competenc* OR monitor* OR surveillance OR (risk N1 assess*) OR (care N1 coordinat*) OR (follow N1 up)) OR AB (nurs* OR "advanced practice" OR implement* OR adherence OR (guideline N1 concordan*) OR (quality N1 indicator*) OR (quality N1 measure*) OR (quality N1 improvement) OR (care N1 pathway*) OR (care N1 gap*) OR (service N1 model*) OR (survivorship N3 plan*) OR referral* OR rehabilitat* OR educat* OR competenc* OR monitor* OR surveillance OR (risk N1 assess*) OR (care N1 coordinat*) OR (follow N1 up)) | 1,941,611 |
| S8 | TI (Asia OR Asian OR (Asia N1 Pacific) OR Oceania* OR Australasia* OR China OR Chinese OR "Hong Kong" OR Taiwan OR Japan* OR Korea* OR India* OR Singapore* OR Malaysia* OR Indonesia* OR Philippine* OR Thailand OR Vietnam* OR Pakistan* OR Bangladesh* OR "Sri Lanka" OR Nepal* OR Australia* OR (New N1 Zealand*) OR (Pacific N1 Island*) OR rural OR (low N1 resource*) OR (resource N1 limit*)) OR AB (Asia OR Asian OR (Asia N1 Pacific) OR Oceania* OR Australasia* OR China OR Chinese OR "Hong Kong" OR Taiwan OR Japan* OR Korea* OR India* OR Singapore* OR Malaysia* OR Indonesia* OR Philippine* OR Thailand OR Vietnam* OR Pakistan* OR Bangladesh* OR "Sri Lanka" OR Nepal* OR Australia* OR (New N1 Zealand*) OR (Pacific N1 Island*) OR rural OR (low N1 resource*) OR (resource N1 limit*)) | 550,076 |
| S9 | S7 OR S8 | 2,304,460 |
| S10 | S6 AND S9 | 784 |
| Limits | English, publication date 2015-01-01 to 2026-07-31 | 641 |

**Supplementary Table S2. Eligibility, classification, and selection criteria.**

| **Domain** | **Operational criterion** |
| --- | --- |
| Population and context | Adults with cancer or survivors, relevant health professionals, and relevant services. Pediatric evidence was used only when it informed long-term adult survivorship. |
| Concept | Guidance, care delivery, nursing roles or education, service barriers, referral, rehabilitation, survivorship, quality measurement, digital workflow, implementation, or resource adaptation. |
| Evidence types | Guidelines, practice statements, quality frameworks, empirical studies, service evaluations, surveys, implementation studies, and directly relevant reviews. |
| Date and language | English-language records from 2015-01-01 through 2026-07-31 in the structured search. Earlier sources were considered only through documented citation checking. |
| Exclusions | No substantive cancer-related cardiovascular care content, mechanistic-only evidence, diagnostic performance without a care-delivery question, a non-transferable case report, an insufficient conference abstract, or a duplicate or superseded version. |
| Final retention judgment | A source was retained when it filled a defined evidence role with sufficient directness or incremental value. Recency, treatment diversity, nursing relevance, and relevance to adaptation were considered. The preliminary relevance category was not binding. |

**Supplementary Table S3A. Source-selection counts and the material documented for revision checks.**

| **Stage or category** | **Count** | **Interpretation** |
| --- | --- | --- |
| Database exports | 7,972 | Records before de-duplication |
| After automated de-duplication | 6,459 | Identifier and normalized-title matching |
| Additional duplicate or version records removed | 40 | Manually reviewed by Chen Chen, Wenjiao Shan, and Jingjing Hu. Item-level human sign-off and dates were not retained |
| Unique title–abstract records classified | 6,419 | Manual classification using prespecified relevance categories after a 100-record calibration among the three authors |
| Directly relevant | 162 | Manual relevance category used to prioritize source review, not a systematic-review inclusion set |
| Contextual or indirect | 548 | Potential background, determinant, or transferability evidence |
| Full-text clarification | 269 | Potential relevance not resolvable from title and abstract |
| Outside scope | 5,440 | Did not meet the preliminary operational criteria |
| Final references | 42 | Author-selected and author-confirmed cited set |
| Matched to the 6,419-record database pool | 30 | Item-level match retained in the screening workbook |
| Documented pre-2015 citation checking | 3 | Refs. 12–14 |
| Original retrieval route not documented | 9 | Refs. 1, 3, 4, 11, 15, 17, 25, 26, and 42. This is a retrieval-record gap |
| Full text available for recorded revision claim checks | 26 | Includes nine full texts examined during revision and the targeted full-text check of ref. 41 |
| Publisher abstract available for the recorded check | 1 | Huang et al., ref. 32 |
| PubMed abstract available for the recorded check | 14 | Use limited to claims supportable from the abstract |
| Index record available for bibliographic checking | 1 | Wang et al., ref. 40. Bibliographic identity only |
| Candidates in the earlier additional verification exercise | 18 | 17 full texts and one publisher abstract. One full-text source was not retained. |

The four preliminary categories sum to 6,419 and prioritized author review. The verification counts describe material documented for claim checks as of October 9, 2026.

**Supplementary Table S3B. Sources, retrieval records, and claim checks documented during revision.**

| **Ref.** | **Source and original retrieval route** | **Database record and preliminary category** | **Evidence type and role** | **Material for the recorded check** | **Claim-use boundary** |
| --- | --- | --- | --- | --- | --- |
| 1 | Bray F (2024) Route: Not documented | No database-pool match Category: Not documented | Global descriptive epidemiology report Role: Global cancer incidence and mortality context | Full text | Supports estimates of nearly 20 million new cancer cases and 9.7 million cancer deaths worldwide in 2022. This is global epidemiologic context, not nursing or cardio-oncology evidence. |
| 2 | Sturgeon KM (2019) Route: Database pool | APJON-03855 Category: Outside scope | Population-based observational study Role: Population-level cardiovascular mortality burden among US patients with cancer | PubMed abstract | Supports population-level cardiovascular mortality background. Does not establish a nursing-care gap or a causal effect of cancer treatment. |
| 3 | Armenian SH (2016) Route: Not documented | No database-pool match Category: Not documented | Retrospective survivor cohort Role: Long-term CVD incidence and overall-survival context in adult-onset cancer survivors | Full text | Applies to at least two-year survivors diagnosed at age 40 years or older in one Southern California integrated health system. It does not establish a nursing gap, a care-delivery failure, CVD-specific mortality causation, or treatment-specific effects. |
| 4 | Lyon AR (2022) Route: Not documented | No database-pool match Category: Not documented | Clinical practice guideline Role: Principal clinical guidance across the cardio-oncology care continuum | Full text | Supports clinical tasks across the care continuum. It is not direct nursing evidence, service-gap evidence, or validation of the preliminary map or P1 to P6. |
| 5 | Armenian SH (2017) Route: Database pool | APJON-02418 Category: Contextual or indirect | Clinical practice guideline Role: Adult cancer-survivor cardiac dysfunction prevention and monitoring guidance | PubMed abstract | Supports the broad prevention and monitoring recommendations stated in the abstract. Detailed regimen-specific requirements rely on the full-text clinical guidance cited alongside it. |
| 6 | Dent S (2026) Route: Database pool | APJON-00475 Category: Contextual or indirect | Clinical practice statement Role: Direct comparison and five clinical stages | Full text | This is a consensus and clinical practice statement, not a formally validated implementation framework. Referral thresholds and role allocation vary by patient, regimen, and local service configuration. For resource-constrained settings, the statement emphasizes lifestyle support and use of primary-care and community resources. |
| 7 | Lee GA (2024) Route: Database pool | APJON-02452 Category: Directly relevant | Nursing narrative review Role: Existing oncology-nurse roles, novelty comparison | Full text | This is primarily a guidance-oriented synthesis. Some role descriptions depend on nursing competencies and specialist support. The HFA-ICOS tool applies only to specified treatment categories. The paper does not formally develop or validate measurable indicators. |
| 8 | Reehal P (2025) Route: Database pool | APJON-02397 Category: Directly relevant | Specialist nursing service description Role: Specialist nurse role and scope boundary | Full text | This report describes one highly specialized UK center. Responsibilities depend on specialist training, a cardiology background, and independent prescribing authority. The same authority cannot be transferred to general oncology nurses. The ARNI audit effect should not be used when its denominator is unclear. |
| 9 | Lee GA (2023) Route: Database pool | APJON-01305 Category: Directly relevant | Formal quality-indicator guidance Role: Formal indicator definitions | Full text | Some indicators rely on expert opinion and reflect European guidance, resources, and feasibility. They are not equivalent to the author-proposed measures in this review. Local data sources and role adaptation are required. |
| 10 | Rao VU (2025) Route: Database pool | APJON-03552 Category: Contextual or indirect | Quality-of-care roadmap Role: Quality-measure roadmap and denominator correction | Full text | The paper states that clearly defined cardio-oncology performance metrics are not yet established. Its measures are a roadmap and candidate directions. Clinician-level measurement generally requires about 30 to 50 events per period for reliable estimates. |
| 11 | Baethge C (2019) Route: Not documented | No database-pool match Category: Not documented | Narrative-review reporting methodology Role: Narrative-review reporting aid | Full text | SANRA supports appraisal and reporting of a narrative review manuscript. It is not a risk-of-bias tool for included sources and does not establish systematic-review completeness. |
| 12 | Proctor E (2011) Route: Citation checking | No database-pool match Category: Not documented | Implementation-outcomes conceptual paper Role: Separate implementation from clinical outcomes | PubMed abstract | Supports the distinction between implementation outcomes and service or clinical outcomes. Does not validate the candidate measures. |
| 13 | Curran GM (2012) Route: Citation checking | No database-pool match Category: Not documented | Hybrid-design methodological paper Role: Rationale for effectiveness-implementation hybrid evaluation | PubMed abstract | Supports the rationale for hybrid effectiveness–implementation designs. Does not establish the feasibility of a specific evaluation. |
| 14 | Armstrong GT (2013) Route: Citation checking | No database-pool match Category: Not documented | Childhood-cancer survivor cohort Role: Long-term cardiovascular risk and modifiable risk-factor context in survivors of childhood cancer | PubMed abstract | Supports persistent cardiovascular risk among adult survivors of childhood cancer. Does not by itself identify a transition-of-care failure. |
| 15 | Damschroder LJ (2022) Route: Not documented | No database-pool match Category: Not documented | CFIR framework update Role: Source for updated 2022 CFIR construct names and definitions | Full text | Supports purposeful selection of updated CFIR determinants and their formal names. It does not validate the map, establish a site-level CFIR assessment, or validate determinant-to-strategy links. |
| 16 | Lyon AR (2020) Route: Database pool | APJON-01533 Category: Contextual or indirect | Risk-stratification consensus Role: Baseline cardiovascular risk stratification framework | PubMed abstract | Supports the purpose of baseline cardiovascular risk stratification. Does not authorize a nursing role or validate the practice map. |
| 17 | Zheng H (2023) Route: Not documented | No database-pool match Category: Not documented | Guideline evidence appraisal Role: ESC recommendation-class and evidence-level denominator clarification | Full text | Supports the denominator-specific appraisal of recommendation class and level of evidence. It is not clinical guidance, nursing evidence, care-gap evidence, or validation of the practice map. |
| 18 | Chavez-MacGregor M (2015) Route: Database pool | APJON-04172 Category: Full-text clarification | Observational monitoring study Role: Historical trastuzumab cardiac-monitoring gap in older adults | PubMed abstract | Supports historical cardiac-monitoring patterns in older patients receiving trastuzumab. Does not estimate current ESC-guideline adherence or nursing performance. |
| 19 | Henry ML (2018) Route: Database pool | APJON-00163 Category: Full-text clarification | Observational monitoring study Role: Historical cardiotoxicity and cardiac-monitoring patterns during breast-cancer chemotherapy | PubMed abstract | Supports historical monitoring and cardiotoxicity patterns during breast-cancer chemotherapy. Does not establish a nursing intervention effect. |
| 20 | Visser A (2016) Route: Database pool | APJON-01504 Category: Directly relevant | Multicenter monitoring study Role: Historical adherence to cardiac-monitoring recommendations during trastuzumab therapy | PubMed abstract | Supports the abstract-reported baseline and follow-up monitoring proportions in a trastuzumab population. These are not a cross-cancer attrition pathway. |
| 21 | Tang M (2021) Route: Database pool | APJON-01579 Category: Full-text clarification | Population-based monitoring study Role: Australian population-based trastuzumab assessment gap and regional context | PubMed abstract | Supports historical Australian baseline and on-treatment assessment patterns during trastuzumab therapy. Does not measure current guideline adherence across Asia-Pacific systems. |
| 22 | Alhuthali A (2023) Route: Database pool | APJON-02284 Category: Directly relevant | Hospital monitoring study Role: Tertiary-hospital trastuzumab monitoring-adherence context | PubMed abstract | Supports monitoring patterns in the reported tertiary-hospital trastuzumab population. Does not establish region-wide adherence or nursing performance. |
| 23 | Lim A (2022) Route: Database pool | APJON-00322 Category: Directly relevant | Hospital surveillance study Role: Korean tertiary-hospital CTRCD surveillance and follow-up context | PubMed abstract | Supports surveillance and follow-up patterns in the reported Korean hospital cohort. Findings are specific to its population and setting. |
| 24 | Cardinale D (2015) Route: Database pool | APJON-01080 Category: Outside scope | Anthracycline cardiotoxicity cohort Role: Timing of detection and treatment in recovery from anthracycline cardiotoxicity | PubMed abstract | Supports the association between earlier detection and heart-failure treatment and recovery after anthracycline cardiotoxicity. Does not establish a universal escalation threshold. |
| 25 | Zullig LL (2023) Route: Not documented | No database-pool match Category: Not documented | Medication-adherence systematic review Role: CVD risk-factor medication-adherence context among cancer survivors | Full text | Supports the relevance of CVD risk-factor medication-adherence problems. The heterogeneous review does not establish a nursing intervention, a universal pathway, a clinical threshold, or patient-outcome benefit. |
| 26 | Campbell KL (2019) Route: Not documented | No database-pool match Category: Not documented | Exercise consensus and guideline Role: Exercise and safety guidance for cancer survivors | Full text | Supports exercise and safety guidance for cancer survivors. It is not service-gap evidence, direct nursing evidence, or evidence that a nursing-led pathway is effective. |
| 27 | Gilchrist SC (2019) Route: Database pool | APJON-00293 Category: Contextual or indirect | Rehabilitation scientific statement Role: Cardio-oncology rehabilitation scientific guidance | PubMed abstract | Supports the broad rationale for cardio-oncology rehabilitation described in the abstract. Does not validate the proposed nursing referral process. |
| 28 | Fadol A (2025) Route: Database pool | APJON-03793 Category: Directly relevant | Nursing learning-needs survey Role: Education needs and corrected item denominator | Full text | The English-language convenience sample was self-selected and used fixed response options. Country representation was highly uneven, and item-level missingness changed denominators. It should not be described as globally or Asia-Pacific representative. Percentages from multiple-response items should not be summed to 100%. |
| 29 | Zhang C (2023) Route: Database pool | APJON-02472 Category: Directly relevant | Provincial nursing knowledge survey Role: China oncology-nurse knowledge and education-needs evidence | Full text | The sample was limited to tertiary hospitals in Shanxi Province. A self-developed instrument and strict all-items-correct scoring may lower domain and total scores. The results should not be presented as the general knowledge level of nurses across China or the Asia-Pacific region. |
| 30 | Kobayashi Y (2025) Route: Database pool | APJON-00701 Category: Directly relevant | Repeated physician surveys Role: Japan post-guideline regional evidence | Full text | Response rates were low and declined over time. The surveys were limited to JGOG gynecologic oncologists and endometrial cancer care. Responses were self-reported, and repeated participation could not be identified. The findings do not represent all Japanese services, nurses, or the wider Asia-Pacific region. |
| 31 | Poley S (2026) Route: Database pool | APJON-01931 Category: Directly relevant | Hospital service survey Role: Contemporary German system evidence | Full text | The study had a low response rate, likely academic-center overrepresentation, self-report and social-desirability bias, and coverage limited to inpatient departments. Item-level denominators differed and missing data were not imputed. The findings do not represent all German services or patient-level guideline adherence. |
| 32 | Huang PL (2026) Route: Database pool | APJON-00642 Category: Directly relevant | Medicare claims analysis Role: Recent publication using pre-guideline data | Publisher abstract | The data cover 2014 to 2018 and predate the 2022 ESC guideline. The study used US Medicare claims from older adults. It cannot be described as measuring contemporary ESC guideline adherence and should not be generalized to all ages, cancers, or health systems. |
| 33 | Haj-Yehia E (2026) Route: Database pool | APJON-01717 Category: Directly relevant | Single-center ICI registry Role: ICI treatment-diversity context | Full text | This was a single-center retrospective study with a heterogeneous sample dominated by melanoma. Definitions and surveillance intensity changed between study periods, creating selection and detection bias. The study cannot show that more intensive monitoring caused either higher or lower toxicity. |
| 34 | Merseburger AS (2024) Route: Database pool | APJON-01527 Category: Directly relevant | Asia-Pacific expert recommendations Role: ADT and Asia-Pacific protocol adaptation | Full text | Expert selection and resource availability may have influenced the recommendations. No Delphi process or formal consensus threshold was reported. The source cannot estimate actual adherence or universal effectiveness across the Asia-Pacific region. |
| 35 | Douglas E (2024) Route: Database pool | APJON-01537 Category: Directly relevant | Anti-estrogen therapy cohort Role: Referral rate requires appropriateness denominator | Full text | This single-center retrospective study is vulnerable to confounding by indication, missing information, and incomplete outside records. Higher referral or testing rates do not necessarily indicate better quality and cannot establish a causal effect of ovarian function suppression. |
| 36 | Clouser JM (2024) Route: Database pool | APJON-03099 Category: Directly relevant | CFIR service-implementation study Role: Direct CFIR-guided service evidence | Full text | This single-center study had a small sample, did not report thematic saturation, and could not calculate a survey response rate. The proposed service was not yet operating in a standardized form. Findings should not be generalized to all systems or treated as nursing-specific barriers. |
| 37 | Weaver KE (2025) Route: Database pool | APJON-01594 Category: Directly relevant | Practice-randomized EHR trial Role: Direct EHR workflow trial, outcome-specific claims | Full text | Only nine practices with sufficient information-technology capacity or interest participated, and the sample was predominantly female and affected by breast cancer. Outcomes were mainly short-term communication and process measures. The study does not show that EHR prompts improved clinical outcomes or recorded referrals. |
| 38 | Fadol A (2026) Route: Database pool | APJON-02081 Category: Directly relevant | Nursing education program description Role: Nursing education program, no clinical outcome | Full text | This is a description of curriculum development and implementation. It has no comparison group and does not yet report clinical or patient outcomes. Availability of the education program should not be described as effectiveness or implementation success. |
| 39 | Chen DH (2026) Route: Database pool | APJON-00035 Category: Directly relevant | Australian service narrative review Role: Australia metropolitan/rural service context | Full text | This is a narrative review. Some estimates come from secondary sources or local programs, and service counts may change over time. The review does not represent the entire Asia-Pacific region and cannot estimate nursing implementation rates. |
| 40 | Wang X (2024) Route: Database pool | APJON-03615 Category: Contextual or indirect | China-focused editorial Role: China field-development context | PubMed index record, no abstract | The index record verifies the editorial’s bibliographic identity and topic only. No substantive clinical or service claim is based on this record. |
| 41 | Kappel C (2022) Route: Database pool | APJON-03334 Category: Directly relevant | Telehealth narrative review Role: Rural telehealth limitations/opportunity | Full text Telehealth claim passages checked during revision | A Canadian-context review and proposed access model. Relevant full-text passages describe connectivity, equipment, physical-examination, and rural testing limitations. It does not demonstrate effectiveness of this practice map or establish transferability to Asia-Pacific settings. |
| 42 | Jacobsen PB (2018) Route: Not documented | No database-pool match Category: Not documented | Survivorship care-plan systematic review Role: Survivorship care-plan effects and outcome uncertainty | Full text | Supports caution that survivorship care-plan delivery alone has uncertain effects. It is not cardiovascular-specific, nursing-specific, or validation of the proposed P6 workflow. |

Reference numbers correspond to the main-text reference list. Retrieval route concerns how a source originally entered the review. Verification material concerns the content available for the recorded claim check. Original preliminary categories are preserved. An abstract-based check supports only statements explicitly reported in that abstract.

**Supplementary Table S3C. The 18 candidates in the additional source-level verification exercise.**

| **No.** | **Source** | **Final status** | **Access level** | **Design or setting** | **Verified use in the review** | **Principal boundary** |
| --- | --- | --- | --- | --- | --- | --- |
| 1 | Chen et al., 2026. The Current Cardio-Oncology Landscape Across Australia | Retained as ref. 39 | Full text | Narrative review of Australian service development and access | Supports metropolitan and rural service-context discussion | Narrative synthesis with time-sensitive service counts. Not an adherence study. |
| 2 | Dent et al., 2026. IC-OS/MASCC clinical practice statement | Retained as ref. 6 | Full text | IC-OS and MASCC clinical practice statement | Anchors the five clinical care stages | Clinical guidance. It does not establish nursing-specific effectiveness. |
| 3 | Huang et al., 2026. Cardiac monitoring variation among older adults | Retained as ref. 32 | Official publisher abstract only | US Medicare claims analysis of 2,143 older adults | Supports treatment-diverse historical screening estimates | Official publisher abstract only. Data were collected in 2014–2018. |
| 4 | Haj-Yehia et al., 2026. ICI cardiac surveillance, Essen registry | Retained as ref. 33 | Full text | Single-center registry of 539 ICI recipients | Supports regimen-specific surveillance context | Selection and detection bias. Not an adherence or nursing study. |
| 5 | Poley et al., 2026. Cardio-oncology care in German hospitals | Retained as ref. 31 | Full text | German hospital survey with 120 of 1,435 departments responding | Supports post-guideline service variation | Low response rate and academic-center overrepresentation. |
| 6 | Fadol et al., 2026. Cardio-Oncology Nursing Education (CONE) | Retained as ref. 38 | Full text | Description of the 27-module CONE education program | Supports availability of a nursing education resource | No patient, service, or implementation outcomes reported. |
| 7 | Ivanescu AC et al. (2025). Current Knowledge and Implementation of Therapeutic Strategies in Cardio-Oncology: A National Physician-Based Survey. American Journal of Therapeutics. PMID 40338679. DOI 10.1097/MJT.0000000000001970 | Checked but not retained | Full text | Romanian national cross-sectional physician survey using a validated 21-item online questionnaire | Not used in the final synthesis. The current appraisal identifies physician knowledge and strategy variation as its potential evidence role. | The historical nonretention rationale was not recorded. Small, cardiology-heavy sample and no reported invitation denominator. |
| 8 | Kobayashi et al., 2025. JGOG surveys in endometrial cancer | Retained as ref. 30 | Full text | Repeated surveys of Japanese gynecologic oncologists | Supports regional post-guideline service context | Low response rates and profession-specific scope. |
| 9 | Weaver et al., 2025. WF-1804CD EHR application trial | Retained as ref. 37 | Full text | Practice-randomized EHR application trial with 627 evaluable survivors | Supports digital workflow and outcome-level distinctions | Selected practices and a largely breast cancer population. No demonstrated cardiovascular clinical benefit. |
| 10 | Reehal et al., 2025. UK cardio-oncology clinical nurse specialist | Retained as ref. 8 | Full text | Description of a UK specialist cardio-oncology clinical nurse service | Supports specialist-role and scope-boundary discussion | Context-specific service description. Not transferable to general nursing scope. |
| 11 | Rao et al., 2025. Quality-of-care measures for cardio-oncology | Retained as ref. 10 | Full text | IC-OS and ACC quality-of-care roadmap | Supports distinction between recommendations and performance measures | Conceptual and methodological guidance, not validation of P1–P6. |
| 12 | Fadol et al., 2025. International nursing learning-needs survey | Retained as ref. 28 | Full text | International English-language nursing learning-needs survey | Supports nursing education needs and the 268 of 305 item denominator | Convenience sample with item-level missingness and North American dominance. |
| 13 | Merseburger et al., 2024. APMA PCCV recommendations for ADT | Retained as ref. 34 | Full text | Asia-Pacific multidisciplinary recommendations for ADT | Supports regimen-specific risk assessment and local pathway adaptation | Expert recommendations. They do not measure regional adherence. |
| 14 | Douglas et al., 2024. Anti-estrogen therapy and CV actions | Retained as ref. 35 | Full text | US cohort of 217 premenopausal patients receiving anti-estrogen therapy | Supports indication-sensitive interpretation of referral and testing | Single-center cohort and selected population. |
| 15 | Lee and Reehal, 2024. Role of the oncology nurse | Retained as ref. 7 | Full text | Narrative review of the oncology nurse role | Supports potential nursing touchpoints across care stages | Not an empirical evaluation of nursing outcomes or local authority. |
| 16 | Clouser et al., 2024. CFIR-guided cardio-oncology service implementation | Retained as ref. 36 | Full text | Single-center CFIR mixed-methods implementation study | Supports service determinants and interpretive CFIR mapping | Thirteen interviewees and 33 analyzable survey responses. Not nursing-specific. |
| 17 | Zhang et al., 2023. Cardio-oncologic knowledge of oncology nurses in China | Retained as ref. 29 | Full text | Multicenter survey of 736 oncology nurses in Shanxi Province | Supports a locally observed nursing knowledge gap | Provincial tertiary-hospital sample and stringent scoring. Not China-wide. |
| 18 | Lee et al., 2023. ESC cardio-oncology quality indicators | Retained as ref. 9 | Full text | Formal ESC cardio-oncology quality indicators | Supports population, numerator, denominator, and period logic | Formal indicators are not equivalent to the author-proposed P1–P6 measures. |

The additional exercise comprised 17 full-text checks and one publisher-abstract check. Huang et al. (ref. 32) was the publisher-abstract source. Ivanescu et al. was checked in full text but was not retained. The scope of this exercise is distinct from the review search and source-selection process.

**Supplementary Table S3D. Evidence roles of five retained sources after reassessment during revision.**

| **Ref.** | **Source** | **Original preliminary category** | **Material for current reassessment** | **Retention rationale recorded during revision** | **Original selection record** |
| --- | --- | --- | --- | --- | --- |
| 2 | Sturgeon KM (2019) | Outside scope in preliminary classification | PubMed abstract | Retained for population-level background on cardiovascular mortality among people with cancer. It does not support a nursing role, a care-delivery pathway, or practice-map effectiveness. | Item-level rationale, decision-maker, and date not retained |
| 18 | Chavez-MacGregor M (2015) | Full-text clarification | PubMed abstract | Retained as historical treatment-specific care-delivery evidence showing variation in cardiac monitoring among older patients receiving adjuvant trastuzumab. It identifies a surveillance failure point but does not attribute the gap to nurses. | Item-level rationale, decision-maker, and date not retained |
| 19 | Henry ML (2018) | Full-text clarification | PubMed abstract | Retained as historical observational evidence on cardiotoxicity and cardiac monitoring among chemotherapy-treated patients with breast cancer. It informs monitoring-gap context, not a nursing-specific intervention. | Item-level rationale, decision-maker, and date not retained |
| 21 | Tang M (2021) | Full-text clarification | PubMed abstract | Retained as Australian population-based evidence on baseline and on-treatment cardiac assessment during trastuzumab therapy. It contributes historical monitoring-gap estimates and regional context, not current ESC adherence or nursing performance. | Item-level rationale, decision-maker, and date not retained |
| 24 | Cardinale D (2015) | Outside scope in preliminary classification | PubMed abstract | Retained as prospective cohort evidence that earlier detection and heart-failure treatment were associated with better recovery after anthracycline cardiotoxicity. It supports timely multidisciplinary response, not a universal trigger or a nurse-led treatment pathway. | Item-level rationale, decision-maker, and date not retained |

These rationales were recorded during revision on October 9, 2026. The original item-level reasons and dates for changing from preliminary classification to final retention were not retained. The current rationales clarify source use without reconstructing those historical decisions.

**Supplementary Table S4. How SANRA informed reporting.**

| **SANRA domain** | **Application in this review** |
| --- | --- |
| Justification of importance | The Introduction states the clinical problem and the practical need to connect heterogeneous evidence to nursing touchpoints and handoffs. |
| Statement of aims | The Abstract and Introduction use one objective: synthesize distinct evidence categories and organize them into a preliminary practice map. |
| Description of search | Databases, platforms, dates, full strategies, restrictions, counts, de-duplication, classification, purposive selection, and source routes are reported. |
| Referencing | Key claims are linked to sources. Tables S3B and S3C distinguish bibliographic identity, the material examined for the revision check, and the claim it can support. |
| Scientific reasoning | Direct nursing evidence, guidance, multidisciplinary care-delivery evidence, service evidence, and author proposals are separated. Study limitations are reported near claims. |
| Appropriate presentation of data | Unrelated percentages are not presented as sequential attrition. Denominators, historical data periods, and non-comparability are explicit. |

**Supplementary Table S5. Evidence-to-map provenance by care stage.**

| **Care stage** | **Clinical or quality guidance (G)** | **Direct nursing evidence (N)** | **Multidisciplinary clinical, care-delivery, service, or implementation evidence (S)** | **Author-proposed translation (A)** |
| --- | --- | --- | --- | --- |
| Pretreatment assessment | Refs. 4–6: indicated baseline care. Ref. 16: risk categories. Ref. 9: measurement logic | Refs. 7 and 8: assessment verification and education activities | Refs. 18–23 and 32: selected historical assessment and monitoring gaps, not evidence of nursing failure | Regimen-linked checklist, assignment of verification responsibilities, and handoff |
| Surveillance during therapy | Refs. 4–6: regimen- and risk-specific monitoring. Ref. 9: measurement logic | Refs. 7 and 8: monitoring follow-up and symptom elicitation | Refs. 18–23 and 32: selected historical monitoring gaps. Ref. 37: digital-prompt analogy only, not direct monitoring-gap evidence | Cycle-linked reminders, completion tracking, and local notification arrangements |
| Toxicity response and risk-factor management | Refs. 4–6: clinical response. Refs. 26 and 27: exercise and rehabilitation guidance | Refs. 7 and 8: symptom elicitation and education | Ref. 24: recovery timing. Ref. 25: medication-adherence barriers. Ref. 33: ICI clinical-risk context, not a general care gap | Structured review, approved education, and notification handoff |
| Referral and rehabilitation | Refs. 4–6, 27, and 34: clinical referral and rehabilitation pathways | Refs. 7 and 8: referral coordination | Refs. 31, 36, and 39: service-access context. Ref. 37: digital outcome distinction. Ref. 41: telehealth limitations | Referral transmission workflow and distinction from downstream access outcomes |
| Survivorship follow-up | Refs. 4–6: long-term follow-up and care-plan requirements | Refs. 7 and 8: education and care coordination | Ref. 14: persistent cardiovascular risk. Ref. 42: uncertain care-plan benefit. Neither establishes a transition failure | Required plan fields, nursing verification, patient and receiving-clinician communication, and follow-up ownership |

This table expands main-text Table 3. G identifies clinical or quality guidance, N direct nursing literature, S multidisciplinary clinical, care-delivery, service, or implementation evidence, and A the authors’ operational translation. Nursing activities described in existing literature are distinguished from the proposed way of combining and measuring them. Roles and clinical decisions remain subject to local authority. Refs. 28, 29, and 38 inform staff education needs or resources, not patient-education effectiveness. Ref. 35 concerns indication-sensitive testing and is not direct evidence of referral-pathway effectiveness.

**Supplementary Table S6. Provenance and adaptation of P1–P6.**

| **Candidate nursing-relevant process measure** | **Established source contribution** | **Author-proposed adaptation** |
| --- | --- | --- |
| P1 Baseline assessment status verification | G: refs. 4–6, 9, and 16 define the clinical requirement, risk framework, and measurement logic. N: refs. 7 and 8 identify possible nursing touchpoints. S: refs. 18–23 and 32 document care-delivery gaps | Regimen-linked checklist, per-element verification statuses, notification of missing items, separate reporting of mixed statuses within a treatment start, and reporting interval |
| P2 On-time protocol-due surveillance completion | G: refs. 4–6 and 9 define clinical surveillance and measurement principles. N: refs. 7 and 8 identify possible nursing touchpoints. S: refs. 18–23 and 32 document delivery gaps | Protocol-due denominator, local completion window, cycle-linked reminders, nursing tracking contribution, and stratified reporting |
| P3 Timely protocol-defined notification of an eligible escalation event | G: refs. 4–6 define clinical concerns and escalation needs. N: refs. 7 and 8 identify possible nursing notification roles. S: refs. 24 and 33 provide timing and treatment-specific context | Single notification endpoint, designated recipient, local interval, and separate downstream clinical-review outcome |
| P4 Cardiovascular education completion | G: refs. 4–6 define the clinical education requirement and content domains. N: refs. 7 and 8 identify possible nursing education roles | Protocol-designated education-encounter denominator, required content set, comprehension or teach-back check, stage-specific eligibility, and documentation fields |
| P5 Timely referral transmission after an authorized decision | G: refs. 4–6, 27, and 34 define referral or rehabilitation pathways. N: refs. 7 and 8 identify possible nursing coordination roles. S: refs. 31, 36, 37, 39, and 41 describe access or workflow barriers | Authorized-decision denominator, transmission endpoint, required information, and separate acceptance, attendance, completion, and alternative-path outcomes |
| P6 Survivorship-plan verification and communication | G: refs. 4–6 define long-term follow-up and care-plan requirements. N: refs. 7 and 8 identify possible nursing contributions. S: refs. 14 and 42 provide risk and care-plan outcome context | Team-agreed plan, required fields, documented nursing verification, communication to the patient and receiving clinician, and follow-up ownership |

This table explains the source contributions to main-text Table 4. The eligibility criteria, numerators, denominators, periods, and data sources are specified in that table. P1–P6 are candidate measures of team-delivered pathways with nursing contributions. They have not been endorsed or validated as quality indicators or individual nursing-performance measures.
